# Krankheitsmodifizierende Therapieansätze bei der Huntington-Krankheit: Blicke zurück und Blicke voraus

**DOI:** 10.1007/s00115-021-01224-8

**Published:** 2021-11-11

**Authors:** Wiebke Frank, Katrin S. Lindenberg, Alzbeta Mühlbäck, Jan Lewerenz, G. Bernhard Landwehrmeyer

**Affiliations:** 1 Klinik für Neurologie, Universität Ulm, Oberer Eselsberg 45/1, 89081 Ulm, Deutschland; 2 Huntington-Zentrum Süd, kbo-Isar-Amper-Klinikum, Taufkirchen (Vils), Deutschland; 3 Klinik für Neurologie und Zentrum für klinische Neurowissenschaften, 1. Medizinische Fakultät, Karlsuniversität, Prag, Tschechien

**Keywords:** Neuroprotektion, *HTT*-mRNA, Antisense-Oligonukleotide, Gentherapie, Krankheitsmodifikation, Neuroprotection, *HTT* mRNA, Antisense oligonucleotide, Gene therapy, Disease modification

## Abstract

Die Huntington-Krankheit (HK) ist die häufigste monogenetische neurodegenerative Erkrankung und kann bereits im präklinischen Stadium zweifelsfrei diagnostiziert werden, zumindest in allen Fällen, bei denen die CAG-Expansionsmutation im Huntingtin-Gen (*HTT*) im Bereich der vollen Penetranz liegt. Wichtige Voraussetzungen für eine früh im Krankheitsprozess einsetzende und deshalb den weiteren Verlauf der Krankheit in klinisch relevanter Weise modifizierende Therapie sind damit gegeben und machen die HK zu einer Modellerkrankung für neuroprotektive Behandlungsansätze. In der Vergangenheit lag der Schwerpunkt auf dem Ausgleich vermuteter Neurotransmitterdefizite (GABA) analog zur Parkinson-Erkrankung und auf klassischen neuroprotektiven Strategien zur Beeinflussung hypothetischer gemeinsamer Endstrecken neurodegenerativer Erkrankungen (z. B. Exzitotoxizität, mitochondriale Dysfunktion, oxidativer Stress etc.). Mit der Entdeckung der krankheitsverursachenden *HTT*-Mutation im Jahr 1993 fokussierte sich die Therapieforschung zunehmend darauf, soweit proximal wie möglich in die pathophysiologische Ereigniskette einzugreifen. Ein wichtiger Ansatzpunkt ist hier die *HTT*-mRNA mit dem Ziel, die Nachproduktion mutierter Huntingtin-Genprodukte zu senken und damit den Körper von deren schädigenden Auswirkungen zu entlasten; zu diesem Zweck sind verschiedene Behandlungsmodalitäten (einzelsträngige DNA und RNA, divalente RNA und Zinkfinger-Repressorkomplexe, oral verfügbare Spleißmodulatoren) entwickelt worden, die sich in der klinischen Prüfung (Phase I–III) oder in späten Stadien der präklinischen Entwicklung befinden. Zudem zeichnet sich ab, dass es möglich sein könnte, die Länge der somatisch instabilen, d. h. über die Lebenszeit v. a. im Hirngewebe zunehmende CAG-Mutation selbst zu beeinflussen und die Progression der HK hierdurch zu bremsen.

## Abstract (en)

Huntington disease (HD) is the most frequent monogenetic neurodegenerative disease and can be unequivocally diagnosed even in the preclinical stage, at least in all individuals in whom the CAG expansion mutation in the huntingtin gene (*HTT*) is in the range of full penetrance. Therefore, important preconditions for an intervention early in the disease process are met, rendering modification of the course of the disease in a clinically meaningful way possible. In this respect, HD can be viewed as a model disorder for exploring neuroprotective treatment approaches. In the past emphasis was placed on the compensation of a suspected neurotransmitter deficit (GABA) analogous to Parkinson’s disease and on classical neuroprotective strategies to influence hypothetical common pathways in neurodegenerative diseases (e.g., excitotoxicity, mitochondrial dysfunction, oxidative stress). With the discovery of the causative *HTT* mutation in 1993, therapeutic research increasingly focused on intervening as proximally as possible in the chain of pathophysiological events. Currently, an important point of intervention is the *HTT* mRNA with the aim of reducing the continued production of mutant huntingtin gene products and thus relieving the body of their detrimental actions. To this end, various treatment modalities (single-stranded DNA and RNA, divalent RNA and zinc finger repressor complexes, orally available splice modulators) were developed and are currently in clinical trials (phases I–III) or in late stages of preclinical development. In addition, there is the notion that it may be possible to modify the length of the somatically unstable CAG mutation, i.e. its increase in the brain during the lifetime, thereby slowing the progression of HD.

Seit der Entdeckung der krankheitsauslösenden Mutation im Huntingtin-Gen (*HTT*) im Jahr 1993 hat die Entwicklung krankheitsmodifizierender Therapien für die Huntington-Krankheit (HK) eine fundamentale Transformation erlebt. Lag der Schwerpunkt in der Vergangenheit auf nachgeschalteten pathophysiologischen Mechanismen („gemeinsame Endstrecke neurodegenerativer Erkrankungen“), steht gegenwärtig die Entlastung des Körpers von schädigenden mutanten Genprodukten und die krankheitsverursachende Mutation selbst im Fokus („krankheitsspezifische, proximale Ansätze“). Aktuelle (prä-)klinische Studien und die Ergebnisse von Phase-I/IIb-Studien begründen die Hoffnung, dass durch einen frühen Eingriff in die Kette pathophysiologischer Ereignisse die bisher unaufhaltsame Progression der Erkrankung gebremst werden könnte.

## Hintergrund

Die HK ist eine autosomal-dominant vererbte neurodegenerative neuropsychiatrische Erkrankung, die im Verlauf zu progredienten motorischen und kognitiven Beeinträchtigungen führt. Sie führt typischerweise im mittleren Lebensalter zu Krankheitszeichen, anhand derer die klinische Phase der HK sicher diagnostiziert werden kann, und im Median nach etwa 15 [39] bis 21 Jahren [22] zum Tod. Mit einer Prävalenz von 10 bis 15 pro 100.000 Einwohner in Deutschland zählt sie zu den häufigsten seltenen Erkrankungen [63].

Die genetische Ursache der HK ist eine instabile Expansion einer CAG-Basentriplett-Sequenz innerhalb des ersten Exons des Huntingtin-Gens (*HTT*) auf Chromosom 4 [35]. Der Normalbereich endet bei 35 CAG-Wiederholungen, eine Expansion von mehr als 39 CAG-Wiederholungen führt im Laufe des Lebens immer zum Ausbruch der Erkrankung (vollständige Penetranz), wobei längere CAG-Wiederholungen mit früherem Krankheitsbeginn extensiveren Beeinträchtigungen (z. B. epileptische Anfälle, Ataxie) und rascherer Progression der Erkrankung assoziiert sind [4]. Mittels eines prädiktiven genetischen Tests können *HTT*-Mutationsträger bereits vor dem Auftreten klinischer Symptome zweifelsfrei identifiziert werden. Infolgedessen könnten neuroprotektive Therapien bereits im präsymptomatischen Stadium eingeleitet werden und so potenziell neuronale Dysfunktion und Nervenzellverlust verhindern [13].

Bereits vor der Entdeckung der *HTT*-Mutation sind zahlreiche zelluläre pathophysiologische Alterationen beschrieben worden, die als Ausgangspunkte für die Entwicklung neuroprotektiver Therapien dienten. Zu den ersten vermuteten Pathomechanismen zählte – in Analogie zum Dopaminmangel im Striatum bei der Parkinson-Erkrankung – ein Neurotransmitterdefizit, im Falle der HK eine Depletion von γ‑Aminobuttersäure (GABA) in den Basalganglien [60] mit der Konsequenz einer GABA-Ersatztherapie oder der Gabe von GABA_B_-Rezeptor-Agonisten, z. B. Baclofen [71]. Inspiriert durch die Ähnlichkeit der striatalen Pathologie der HK und den pathologischen Veränderungen nach Applikation von Exzitotoxinen [15], insbesondere von N‑Methyl-D-Aspartat(NMDA)-Rezeptor-Agonisten wie Quinolinsäure [8] oder selektiven Inhibitoren der Atmungskette (3-Nitroproprionsäure, 3NP; [7, 51]), wurden Therapieansätze zur Hemmung der Exzitotoxizität, Förderung der Mitochondrienfunktion und Senkung des zellulären oxidativen Stresses entwickelt. Die Modulation dieser Pathomechanismen war das Ziel zahlreicher präklinischer Studien und klinischer Medikamentenprüfungen (Tab. 1).

Seit der Entdeckung intranukleärer und zytoplasmatischer Proteinaggregate aus polyglutaminexpandierten HTT-Fragmenten im Gehirn transgener Modelle [17] und verstorbener HK-Patienten [18] rückten die proteinopathischen Aspekte der HK und insbesondere den Prozess der Bildung unlöslicher Proteinaggregate in den Vordergrund. In den vergangenen Jahren standen RNA-Ansätze („gene-silencing“ bzw. „huntingtin-lowering“) im Mittelpunkt des Interesses. In jüngster Zeit konzentrieren sich viele Therapieansätze darauf, die Länge der somatisch instabilen CAG-Mutation, deren Ausprägung im Laufe des Lebens zunimmt [37] selbst zu beeinflussen und zwar durch Modulation von DNA-Reparaturvorgängen, die zu einer weiteren Expansion der CAG-Mutation in einer gewebespezifischen Weise führen („somatische Instabilität“).

**Table 1:** 

Sponsor (Studie)	Agens	Verabreichung	Mechanismus	Allelselektiv	Identifier	Phase	Endpunkt erreicht
**Neurotransmitterersatz**							
IIT	Baclofen	Oral	GABA_B_-Rezeptor-Agonist	–	–	–	Nein [71]
**Exzitotoxizität**							
UBC (Mitigate-HD)	Memantin	Oral	NMDA-Rezeptor-Antagonist	–	NCT01458470	II	Nein
HSG	Riluzol	Oral	Antiexzitotatorisch	–	–	III	Nein [31]
IIT (EHDI)	Riluzol	Oral	Antiexzitotatorisch	–	NCT00277602	III	Nein [46]
IIT	Lamotrigin	Oral	Natriumkanalblocker	–	–	–	Nein [43]
IIT	Amantadin	Oral	NMDA-Rezeptor-Antagonist	–	–	–	Nein [50]
HSG (CAREHD)	Remacemid	Oral	NMDA-Rezeptor-Antagonist	–	–	–	Nein [33]
**Mitochondriale Funktion und oxidativer Stress**							
HSG (CAREHD)	Koenzym Q10	Oral	Mitochondriale Funktion ↑ Oxidativer Stress ↓	–	–	III	Nein [33]
IIT	Ethyl-EPA	Oral	Mitochondriale Funktion ↑	–	–	–	Nein [62]
HSG (2CARE)	Koenzym Q10	Oral	Mitochondriale Funktion ↑ Oxidativer Stress ↓	–	NCT00608881	III	Nein [53]
MGH (CREST-E)	Kreatin	Oral	ATP-Level ↑	–	NCT00712426	III	Nein [30]
NCCIH (CREST-HD)	Kreatin	Oral	ATP-Level ↑	–	NCT00026988	I/II	Nein [29]
IIT	Cysteamin	Oral	BDNF-Spiegel ↑	–	NCT02101957	II/III	Nein [82]
HSG (TREND-HD)	Ethyl-EPA	Oral	Mitochondriale Funktion ↑	–	NCT00146211	III	Nein [20, 34]
**Neuroinflammationsmodulation**							
HSG	Minocyclin	Oral	Antiinflammatorisch	–	NCT00277355	II/III	Nein [32]
Teva (Legato-HD)	Laquinimod	Oral	Inflammationsmodulation	–	NCT02215616	II	Nein [64]
Vaccinex (SIGNAL)	Pepinemab (VX15)	i.v.	Anti-Semaphorin 4D monoklonaler Antikörper	–	NCT02481674	II	Nein [81]
**RNA-Ansätze**							
*ASO*							
Ionis Pharmaceuticals	RG6042	i.th.	*HTT*-mRNA	Nein	NCT02519036	I/IIa	Ja [77]
Wave Life Sciences (Precision-HD1)	WVE-120101	i.th.	m*HTT*-mRNA (SNP1, rs362037)	Ja	NCT03225833	I/II	Nein [84]
Wave Life Sciences (Precision-HD2)	WVE-120102	i.th.	m*HTT*-mRNA (SNP2, rs362331)	Ja	NCT03225846	I/II	Nein [84]

*ASO* Antisense-Oligonukleotide, *ATP* Adenosintriphosphat, *BDNF* „brain-derived neurotrophic factor“, *GABA* γ‑Aminobuttersäure, *EHDI* European Huntington’s disease Initiative, *EPA* Eicosapentaensäure, *HTT* Huntingtin, *IIT* „investigator initiated trial“, *i.th.* intrathekal, *i.v.* intravenös, *MGH* Massachusetts General Hospital, *NCCIH* National Center for Complementary and Integrative Health, *NMDA* N‑Methyl-D-Aspartat, *SNP* Einzelnukleotidpolymorphismus, *UBC* University of British Columbia

## Ein Blick zurück – die „gemeinsame Endstrecke“ neurodegenerativer Erkrankungen als Therapieansatz

Historisch gesehen basierten das Verständnis der Pathogenese und damit auch die ersten krankheitsmodifizierenden Therapieansätze auf Erkenntnissen, die sich aus neuropathologischen und neurochemischen Analysen des Hirngewebes verstorbener HK-Patienten ergaben. Diese wiesen u. a. auf eine selektive neuronale Degeneration GABAerger Projektionsneuronen („medium spiny neurons“) im Striatum hin [83]. Dies führte zu einem besseren Verständnis der pathophysiologischen Grundlagen für die HK-typische Bewegungsstörung [1] und zur Entwicklung pharmakologischer Strategien zum Ausgleich des GABA-Defizits. Eine randomisierte, kontrollierte Prüfung („randomized clinial trial“, RCT) von Baclofen, einem GABA_B_-Rezeptor-Agonist, konnte die erhoffte Wirksamkeit jedoch nicht nachweisen [71].

### Exzitotoxitität.

Intensiv ist versucht worden, die Mechanismen zu verstehen, die zum krankheitstypischen selektiven Neuronenverlust im Striatum führen. Bereits vor Entdeckung der *HTT*-Mutation waren exzitotoxische Mechanismen als Ursache der striatalen Schädigung und der selektiven neuronale Degeneration im Striatum durch eine Dysregulation der exzitatorischen (i.e. glutamatergen) Neurotransmission und somit durch Exzitotoxitität vermutet worden [67]. Passend zu dieser Hypothese wurden von einzelnen Arbeitsgruppen bei HK-Patienten erhöhte Glutamatkonzentrationen im Liquor [59] und Striatum [78] sowie eine reduzierte gliale Glutamattransporteraktivität [9] gefunden. Da im Besonderen eine Aktivierung extrasynaptischer NMDA-Rezeptoren zum Zelltod führen kann [57], folgerte man, dass die bei der HK vermutete Exzitotoxizität primär durch diese vermittelt wird und die Selektivität des Neuronenverlusts möglicherweise durch die spezifischen Expressionsmuster von NMDA-Rezeptor-Untereinheiten auf den unterschiedlich vulnerabelen striatalen Neuronenpopulation bedingt ist [44, 47].

Therapieansätze richteten sich somit auf antiglutamaterg wirkende Substanzen, die die glutamaterge Neurotransmission entweder postsynaptisch (z. B. durch die Blockade von NMDA-Rezeptoren) oder präsynaptisch (z. B. durch die Hemmung von Natriumkanälen) modulieren und so eine Exzitotoxizität hemmen sollten. Auf diesem Ansatz basierten großangelegte klinische Studien mit antiglutamaterg wirkenden Substanzen wie Remacemid [33], Amantadin [50] und Memantin [12] oder mit Riluzol [46] und Lamotrigin [43]. Leider konnte jedoch in keiner dieser RCTs der Krankheitsverlauf der HK klinisch relevant beeinflusst werden (Tab. 1).

### Oxidativer Stress und mitochondriale Funktion.

Die Beobachtung, dass die intrastriatale Injektion von Mitochondrientoxinen ebenfalls zu einem HK-ähnlichen selektiven Neuronenverlust führt [6], stützte die Annahme, dass eine mitochondriale Dysfunktion ein wesentlicher Bestandteil der HK-Pathogenese sein könnte. So zeigt z. B. die intrastriatale Injektion von 3‑Nitropropionsäure bei Nagetieren eine ähnliche neurochemische und histologische Pathologie wie bei der HK [80]. Therapeutisch wurden deshalb in RCTs die Wirksamkeit solcher Nahrungsergänzungsmittel und Antioxidanzien exploriert, von denen man sich versprach, dass diese die mitochondriale Funktion verbessern. Hierzu wurden u. a. Koenzym Q10 [33, 53], Ethyl-Eicosapentaensäure (EPA; [20]) und Kreatin [30] auf Sicherheit, Verträglichkeit und Wirksamkeit überprüft. Eine neuere Studie untersucht, ob Resveratrol, ein antioxidativ wirksames Polyphenol, die Hirnatrophie bei Patienten in frühen Stadien der HK verlangsamen kann (NCT02336633, REVHD). In keiner dieser Studien zeigten sich die erhofften klinischen Effekte (Tab. 1).

### Neuroinflammation.

Wie bei den meisten neurodegenerativen Erkrankungen finden sich auch bei der HK Hinweise auf eine neuroinflammatorische Komponente, z. B. eine Akkumulation aktivierter Mikrogliazellen im Hirngewebe [68]; auch erhöhte Spiegel proinflammatorischer Zytokine wurden berichtet [13]. Daher wird eine Modulation der neuroinflammatorischen Komponente der HK als explorationswürdiger Ansatz angesehen. Eine randomisierte, placebokontrollierte Phase-II-Studie bei HK-Patienten in frühem Stadium der Erkrankung mit dem Immunmodulator Laquinimod (Legato-HD; NCT02215616), die noch nicht vollständig publiziert ist, konnte allerdings keine Verbesserung des klinischen Phänotyps und keine Verlangsamung der klinischen Krankheitsprogression nachweisen; es wurde allerdings eine Verlangsamung der progressiven Hirnatrophie, v. a. im Kaudatum beschrieben [64].

Vaccinex führte eine randomisierte, placebokontrollierte Phase-II-Studie mit einem monoklonalen Antikörper gegen Semaphorin 4D (SEMA4D; VX15) bei Patienten im Frühstadium der HK durch (SIGNAL; NCT02481674). SEMA4D ist ein an neuroinflammatorischen Signalwegen beteiligtes Transmembranprotein; präklinische Studien legen in einem transgenen Mausmodell für die HK nahe, dass VX15 Einfluss auf die Gehirnatrophierate haben könnte. Im Oktober 2020 wurde bekanntgegeben, dass die Studie die primären Endpunkte nicht erreichen konnte (Tab. 1; [81]).

### Pridopidin.

Agonisten des Sigma-1-Rezeptors (SIG1R) könnten über eine Reihe von Mechanismen neuroprotektiv wirken, z. B. durch eine Absenkung der mit der Proteinproduktion verknüpften Belastung des endoplasmatischen Retikulums („ER-Stress“; [13]). Pridopidin (ACR16) ist ein oral verfügbarer, putativer SIG1R-Agonist, der in mehreren präklinischen Studien untersucht worden ist [70]. In drei RCTs bei Patienten mit der HK (HART, MermaiHD, PRIDE-HD) zeigte sich zwar keine robuste Verbesserung der motorischen Symptome; allerdings ergab sich eine Tendenz zu einem geringeren Funktionsverlust („total functional capacity“, TCF) bei langer Behandlungsdauer (≥ ein Jahr; [52, 65]). Ende 2020 wurde daher eine groß angelegte, randomisierte, placebokontrollierte Phase-III-Studie zur Untersuchung der Wirksamkeit von Pridopidin begonnen (Proof-HD, NCT04556656).

Zusammenfassend waren die bisher durchgeführten Therapiestudien, die auf eine Modulation der (postulierten) gemeinsamen Endstrecke neurodegenerativer Erkrankungen ausgerichtet waren, nicht erfolgreich (Tab. 1).

## Blicke voraus – „proximale“ Therapieansätze

**Figure 1:**
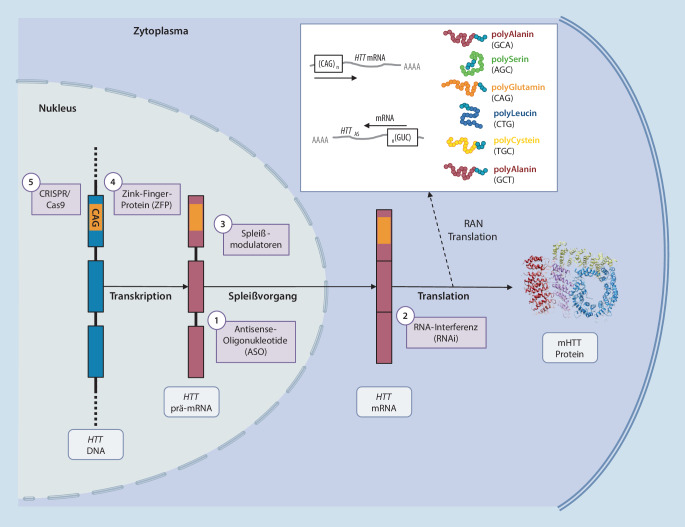

Das tiefere Verständnis der Pathogenese der HK haben in der jüngeren Vergangenheit zu einer Strategieänderung in der Entwicklung krankheitsmodifizierender Therapien geführt. Wie bei den meisten dominanten Erbkrankheiten muss auch bei der HK davon ausgegangen werden, dass die Expansionsmutation und die mutanten Genprodukte neue, auf zellulärer Ebene toxisch wirkende Eigenschaften erwerben („toxic gain-of-function“). Die Idee ist dabei, den Körper von schädigenden Genprodukten (prä-mRNA, reife mRNA, Protein) zu entlasten, wodurch auch alle nachgeschalteten zellulären und systemischen Mechanismen, die möglicherweise zu einer Funktionsbeeinträchtigung führen, abgeschwächt werden sollten. Eine Absenkung der intrazellulären HTT-Spiegel kann auf verschiedenen Wegen erreicht werden, die unterschiedliche Ansatzpunkte haben (Abb. 1).

### Proteinansätze

Ein möglicher proximaler Therapieansatzpunkt ist das mutierte Huntingtin-Protein (mHTT). Es ist bekannt, dass Fragmente des mHTT im Zellkern und Zytoplasma aggregieren [17, 18]. Zusätzlich wird vermutet, dass bei der HK die neuronale Proteostase („zelluläres Proteinequilibrium“) beeinträchtigt ist. Dies könnte zu einem mangelhaften mHTT-Abbau durch das Ubiquitin-Proteasom-System (UPS) bzw. über Autophagie führen und somit zu einer Akkumulation von mHTT. Aufgrund der postulierten neurotoxischen Wirkung der akkumulierenden mHTT-Fragmente erscheinen Therapieansätze Erfolg versprechend, die die zellulären Abbauwege für das mHTT aktivieren [76, 88].

„Intrabodies“ können die Toxizität und Aggregation von mHTT reduzieren

Präklinische Studien mit kleinen, die mHTT-Proteostase beeinflussenden Molekülen zeigten in vitro und in vivo mäßige Effekte und wurden noch nicht in klinischen Studien untersucht [28]. Ein anderer Ansatz besteht in der Verwendung von PROTACs („proteolysis-targeting chimera proteins“) zur gezielten Ausrichtung des UPS auf die Beseitigung von mHTT-Fragmenten. In HK-Fibroblasten konnte z. B. mittels PROTACS die mHTT-Konzentration verringert werden [79]. Ein weiterer Ansatz besteht in der Hochregulierung der autophagischen Proteindegradation. Studien in HK-Zell- und Tiermodellen konnten nachweisen, dass dies zu einer Reduktion der mHTT-Aggregate führt [88]. Andere präklinische Studien zeigten, dass hochspezifische intrazelluläre Anti-HTT-Antikörper („intrabodies“) die Toxizität und Aggregation von mHTT reduzieren können. Ein intrastriatal injizierter Adeno-assoziierter Virusvektor (AAV-Vektor) mit der Kodierungssequenz für die INT41-Intrabodies (rAAV6-INT41) konnte die striatalen HTT-Aggregate reduzieren und die Kognition im R6/2-Mausmodell der HK verbessern [3]. Es gibt zudem Hinweise, dass mHTT von Zelle zu Zelle weitergegeben werden kann und mHTT sich so ähnlich wie Prionen im Gewebe ausbreiten könnte [58]. Daher könnte eine Immuntherapie, beispielsweise mit monoklonalen Antikörpern, zur Bindung und Degradation des mHTT während der extrazellulären Transitphase die Ausbreitung toxischer mHTT-Fragmente vermindern.

### „Gene-silencing“-, „Huntingtin-lowering“-Ansätze

Weitere proximale Behandlungsansätze sind darauf ausgerichtet, die Nachproduktion mutierter Huntingtin-Genprodukte zu senken und damit den Körper von schädigenden Genprodukten zu entlasten.

### mRNA-basierte Ansätze

Eine Vielzahl von Ansätzen zur Reduktion der *HTT*-mRNA wurde in (prä-)klinischen Studien untersucht. Im Vordergrund stehen Antisense-Oligonukleotide (ASOs), RNA-Interferenz-basierte Strategien (RNAi) und orale Spleißmodulatoren [85]. Jeder dieser Ansätze interveniert auf der RNA-Ebene (Abb. 1) und kann in vitro und in Tiermodellen die HTT-Spiegel senken. Im Hinblick auf die klinische Erprobung stellen ASOs die am weitesten entwickelte Strategie dar (Tab. 2).

**Table 2:** 

Sponsor (Studie)	Agens	Verabreichung	Mechanismus	Allelselektiv	Identifier	Phase
**Attenuierung von Pathomechanismen**						
*SIG1R-Agonist*						
Prilenia (Proof-HD)	Pridopidin	Oral	Oxidativer Stress ↓ BDNF-Spiegel ↑	–	NCT04556656	III
**RNA-Ansätze**						
*ASO*						
Hoffmann-La Roche (GENERATION-HD1)	RO7234292	i.th.	prä-mRNA	Nein	NCT03761849	III
Hoffmann-La Roche (GenExtend)	RO7234292	i.th.	prä-mRNA	Nein	NCT03842969	Open-label-Extension
Wave Life Sciences (Precision-HD3)	WVE-003	i.th.	prä-mRNA (SNP3)	Ja	n.n.	I/II
*RNA-Interferenz*						
uniQure	rAVV5-miRNA (AMT-130)	i.c.	mRNA	Nein	NCT04120493	I/II
Spark/Chop	AAV1-miRNA	i.c.	mRNA	Nein	n.n.	Präklinisch
*Spleißmodulatoren*						
PTC Therapeutics	PTC-518	Oral	prä-mRNA	Nein	n.n.	Präklinisch
Novartis	Branaplam	Oral	prä-mRNA	Nein	n.n.	Präklinisch/I
**DNA-Ansätze**						
*Zinkfinger-Protein*						
Sangamo/Shire/Takeda	rAAV-ZFP-RD	i.c.	mRNA	Ja	n.n.	Präklinisch

*ASO* Antisense-Oligonukleotid, *BDNF* „brain-derived neurotrophic factor“, *i.c.* intrazerebral, *i.th.* intrathekal, *HTT* Huntingtin, *miRNA* microRNA, *(r)AVV* (rekombinanter) Adeno-assoziierter Virusvektor, *rAVV-ZFP-RD* rAVV mit Zinkfinger-Protein und Repressordomäne, *SNP* Einzelnukleotidpolymorphismus

#### Antisense-Oligonukleotide

Antisense-Oligonukleotide (ASOs) sind synthetische, einzelsträngige DNA-Moleküle, die entsprechend dem Watson-Crick-Basenpaarungsprinzip an komplementäre (prä-)mRNA-Sequenzen binden. Das daraus hervorgehende RNA-DNA-Hybrid kann, je nach Komposition und Sequenz des ASOs, entweder (1) den Abbau der Ziel-RNA mittels Ribonuklease H1 (RNase H1), (2) die Veränderung des Spleißprozesses oder (3) die Hemmung der Translation bewirken. Der Großteil der bislang entwickelten ASOs wirkt über den zuerst genannten Abbaumechanismus [48] und ist für allelselektives und nichtallelselektives *HTT*-Silencing geeignet. Folglich verringern ASOs die *HTT*-Expression, indem sie die Menge an *HTT*-mRNA verringern und so die Produktion von mHTT vermindern [48, 72].

ASOs können die Blut-Hirn-Schranke nicht passieren

Es gibt jedoch auch einige Herausforderungen. ASOs erzielen nur eine vorübergehende Senkung von mHTT (etwa 3 bis 4 Monate), da sie von körpereigenen Nukleasen abgebaut werden. ASOs müssen intrathekal verabreicht werden, da sie die Blut-Hirn-Schranke nicht passieren können. Diese zeitlich begrenzte Wirksamkeit bietet andererseits den Vorteil, dass die ASO-Therapie jederzeit abgebrochen bzw. pausiert werden kann, sollten sich Unverträglichkeiten zeigen, bedeutet jedoch auch, dass eine wiederholte Verbreichung notwendig ist [23]. Dies stellt für Patienten und Versorgungsstruktur eine erhöhte und andauernde Beanspruchung dar. Eine weitere Herausforderung ist die Verteilung der ASOs im Gehirn. Eine präklinische Studie mit Primaten zeigte nach intrathekaler ASO-Injektion zwar eine nachhaltige *HTT*-mRNA-Senkung in den meisten Hirn- und Rückenmarksregionen; das Ausmaß war jedoch in oberflächlichen Kortex- und Rückenmarksregionen größer als in tieferen Hirnstrukturen (z. B. den Basalganglien; [13, 42]). Sowohl nichtallelselektive als auch allelselektive ASOs werden derzeit bereits in klinischen Studien erprobt (Tab. 2).

##### Nichtallelselektive ASOs.

Das nichtallelselektive *HTT*-ASO (Tominersen – RO7234292) von Roche, dass aktuell in einer randomisierten, placebokontrollierten Phase-III- (GENERATION HD1; NCT03761849) und Open-label-extension-Studie (GenExtend; NCT03842969) erprobt wird, wurde ursprünglich von Ionis Pharmaceuticals Inc. (IONIS-HTT_Rx_) entwickelt. In einer randomisierten, placebokontrollierten Phase-Ib/IIa-Studie konnte nachgewiesen werden, dass alle Dosierungen gut vertragen wurden und zu einer dosisabhängigen Senkung des mHTT-Spiegels im Liquor führten [77]. In GENERATION HD1 wurden mehr als 800 Teilnehmer mit klinisch manifester HK zwei Behandlungsarmen (intrathekale Bolusinjektionen von 120 mg Tominersen, entweder alle 8 oder alle 16 Wochen für 25 Monate) oder einem Placeboarm zugeteilt. Im März 2021 gab Roche auf Empfehlung des unabhängigen Datenüberwachungskomitees (Data and Safety Monitoring Committee, DSMC) den Stopp aller ASO-Injektionen in der Phase-III-Studie bekannt. Eine Analyse des DSMC hatte ergeben, dass Patienten im höheren Dosisarm einen ungünstigeren klinischen Verlauf hatten als Patienten im Placeboarm; zudem zeigte sich eine expositionsabhängige Vergrößerung der inneren Liquorräume. Auch für die laufende offene Nachbehandlungsstudie wurde die ASO-Injektion pausiert. Alle Teilnehmer werden aktuell weiterhin entsprechend dem Protokoll beobachtet. Eine abschließende Entscheidung, ob (und wenn ja, mit welcher Dosis) die klinische Testung mit Tominersen fortgesetzt wird, steht noch aus [66].

Eine hohe Tominersenexposition scheint die zerebralen Ventrikelräume zu erweitern

Ungeklärt ist aktuell, ob die expositions- bzw. dosisabhängigen unerwünschten Effekte der intrathekalen Tominersen(IT)-Gaben auf klassenspezifische Effekte der chemisch modifizierten einzelsträngigen DNA oder auf „On-target“-Effekte zurückzuführen sind. Die wiederholte intrathekale Gabe von Nusinersen – wie auch von Tominersen – scheint zu einer Erweiterung der zerebralen Ventrikelräume bis hin zur Ausbildung eines klinisch apparenten Hydrozephalus führen zu können [10, 75], vermutlich als Folge einer leichtgradigen, sterilen inflammatorischen Reaktion mit konsekutiver Liquorabflussstörung, die sich ab einem bestimmten Ausmaß klinisch negativ auswirkt. Zum anderen könnte eine zu starke Reduktion von HTT, im Besonderen des nichtmutierten Proteins, unerwünschte klinische Effekte nach sich ziehen („On-target“-Effekt).

Da bislang noch ungeklärt ist, ob die nichtallelselektive *HTT*-Reduktion durch Substanzen wie Tominersen die Expression des normalen *HTT*-Allels in bedenklicher Weise absenkt, ist die Entwicklung allelselektiver ASOs ein komplementärer Therapieansatz, der bereits in klinischen Studien erprobt wird.

##### Allelselektive ASOs.

Seit 2017 wird die Sicherheit und Verträglichkeit zweier ebenfalls intrathekal applizierter, allelselektiver ASOs untersucht, entwickelt von der Firma Wave Life Sciences (WVE-120101 und WVE-120102). Gezielt soll nur die mutante (prä-)mRNA für *HTT* abgebaut werden. Dazu werden Genvarianten („single nucleotide polymorphisms“, SNPs) genutzt, die exklusiv auf dem mutierten *HTT*-Allel vorhanden sind [61]. Da sich SNPs in verschiedenen ethnischen Gruppen unterscheiden, wird nicht jeder Patient von einer solchen Therapie profitieren können. Die in den randomisierten, placebokontrollierten Phase-Ib/IIa-Studien Precision-HD1 (NCT03225833) und Precision-HD2 (NCT03225846) verwendeten SNPs sind weltweit bei ca. 50 % bzw. 40 % aller HK-Mutationsträgern vorhanden [61]. Beide Studien wurden abgebrochen, da mit den verwendeten ASOs keine Senkung des mHTT-Spiegels im Liquor erzielt werden konnte [84].

Eine weitere randomisierte, placebokontrollierte Phase-I/II-Studie (Precision-HD3), die ein modifiziertes, potenteres allelselektives ASO (WVE-003) für einen weiteren SNP verwendet, der bei etwa 40 % aller Mutationsträgern vorkommen soll [41], hat im Sommer 2021 begonnen.

#### RNA-Interferenz-basierte Strategien

Weitere vielversprechende Therapieansätze für die HK stellen die RNA-Interferenz(RNAi)-basierten Behandlungsstrategien dar. RNAis sind, wie ASOs, nukleotidbasierte Moleküle, die jedoch an reife, d. h. gespleißte mRNA im Zytoplasma binden und nach Bildung eines RNA-RNA-Duplex im „RNA-induced silencing complex“ (RISC) abgebaut werden [85]. Zu den RNAi-Molekülen gehören „small interfering RNAs“ (siRNAs), „short hairpin RNAs“ (shRNAs) und „microRNAs“ (miRNAs); auch hier gibt es allelselektive und nichtallelselektive Behandlungsansätze.

shRNA und miRNA, verpackt in Genfähren, werden stereotaktisch direkt ins Hirnparenchym eingebracht

Die Grundlage für eine zukünftige allelselektive RNAi-Therapie für die HK wurde u. a. von Pfister und Kollegen [61] gelegt. Die polaren RNAi-Moleküle gelangen bei intrathekaler Injektion nicht gut ins Hirngewebe, weshalb für shRNA und miRNA eine stereotaktische Verabreichung direkt ins Hirnparenchym mittels Genfähren (modifizierter Adeno-assoziierter Virus(AAVs)- [74, 85] oder lentiviraler Vektoren [11]) erforderlich ist. UniQure entwickelte eine rekombinante AAV-basierte Therapie (rAAV5-miHTT; AMT-130), die eine speziell für die Bindung an *HTT*-Exon 1 entwickelte miRNA exprimiert und so zu einer nichtallelselektiven Reduktion der *HTT*-mRNA führen soll [54]. Dieser Ansatz erwies sich in mehreren In-vitro- [38] und HK-Tiermodellen als verträglich und führte zu einer mHTT-Reduktion im Gewebe [19, 73]. AMT-130 wurde 2019 von der US Food and Drug Administration (FDA) zur klinischen Erprobung zugelassen; Phase-I/II-Studien zur Sicherheit und Verträglichkeit haben in den USA begonnen (HD.GeneTRX1, NCT04120493); im zweiten Halbjahr 2021 hat die Rekrutierung für eine europäische Open-label-Studie (HD.GeneTRX2) begonnen (Tab. 2).

##### Divalente siRNA.

Ein Abbau von *HTT*-mRNA kann auch mittels divalenter, lipophiler siRNA (di-siRNA) erfolgen. Wie ASOs werden di-siRNA intrathekal verabreicht, scheinen allerdings eine besonders extensive, großräumige Verteilung zu erlauben, sodass das gesamte Zentralnervensystem (ZNS) abgedeckt werden könnte [2]. Divalente siRNA binden an zytoplasmatische, gespleißte („reife“) mRNA; die daraus resultierenden siRNA/mRNA-Doppelstränge werden über RISC abgebaut. Bei transgenen HK-Mäusen (und bei Primaten) kann eine anhaltende Senkung von m*HTT*-mRNA und mHTT im ZNS nachgewiesen werden [2]. Di-siRNAs bieten somit ein großes Potenzial als RNAi-basierte Therapie.

#### Orale Spleißmodulatoren

Auch oral verfügbare, niedermolekulare Spleißmodulatoren wirken über einen gesteigerten Abbau von *HTT*-mRNA. Im Gegensatz zu RNAis und ASOs erlauben Spleißmodulatoren eine Behandlung des gesamten Körpers, nicht nur des ZNS. Zudem stellt eine orale Gabe im Vergleich zu einer intrakraniellen oder wiederholten intrathekalen Verabreichung eine wesentlich weniger invasive und für die Versorger praktikablere Behandlungsoption dar [76].

Mit oral verfügbaren Spleißmodulatoren kann der ganze Körper behandelt werden

Spleißmodulatoren modifizieren sterisch definierte intronische Sequenzen in der nukleären prä-mRNA in einer Weise, dass diese nicht ausgespleißt werden und ein Pseudoexon mit einem vorzeitigen Stoppcodon entsteht. Die translationsassoziierten Qualitätskontrollprozesse der Zelle detektieren das vorzeitige Stoppcodon und leiten den Abbau des fehlerhaften Transkripts ein („nonsense-mediated mRNA decay“; [45]). Um sie bei der HK einsetzen zu können, wurden mittels mHTT-exprimierenden humanen Zellen kleine Moleküle identifiziert [76], die den Spleißvorgang beeinflussen und den (m)HTT-Spiegel auch in vivo senken könnten [40]. Die Therapieeffekte wären reversibel, sodass eine Behandlung bei unerwünschten Wirkungen abgebrochen werden kann. Allerdings besteht aufgrund der geringeren Spezifität ein erhöhtes Risiko von Nebenwirkungen („off-target effects“; [76]).

Spleißmodulatoren wurden auch für die Anwendung bei anderen neurodegenerativen Erkrankungen, z. B. der spinalen Muskelatrophie (SMA), entwickelt. Beispielweise wurde Risdiplam (RG7916) in mehreren klinischen Zulassungsstudien bei der SMA untersucht und im August 2020 von der FDA für die Behandlung bei Kindern und Erwachsenen zugelassen. Branaplam (LMI070), ein von Novartis ursprünglich für die SMA entwickelter Spleißmodulator, soll bei der HK untersucht werden, da in präklinischen Studien gezeigt wurde, dass Branaplam den mHTT-Spiegel senkt und in einer aktuellen Phase-I-Studie (NCT02268552) zudem die *HTT*-mRNA bei SMA-Patienten reduziert hat. Für Ende 2021 plant Novartis daher eine Phase-IIb-Studie zur Untersuchung der Sicherheit und Verträglichkeit von Branaplam (HTT-001) bei erwachsenen HK-Patienten [56].

### DNA-basierte Ansätze

Weitere vielversprechende Behandlungsoptionen setzen direkt an dem krankheitsverursachenden *HTT* an. Entweder über

- die Modulation der *HTT*-Transkription oder
- die direkte Modifikation der *HTT*-DNA-Sequenz (Genomeditierung).

Die Arbeitshypothese ist, dass die Inaktivierung des m*HTT* die Krankheitsprogression stoppen könnte [87]. Beide Ansätze befinden sich derzeit noch in der präklinischen Erprobung.

#### Transkriptionsmodulation mittels Zinkfinger-Protein-Transkriptionsfaktoren

Synthetische Zinkfinger-Proteine (ZFP) können spezifische DNA-Sequenzen erkennen, an diese binden und deren Transkription verhindern und so auch die *HTT*-Expression reduzieren. Sie können so konstruiert werden, dass sie die expandierten CAG-Wiederholungen im mutierten *HTT*-Allel erkennen, und ermöglichen somit eine allelselektive Hemmung der *HTT*-Transkription [13]. Präklinisch konnte nachgewiesen werden, dass allelselektive ZFP-Transkriptionsfaktoren (ZFP-TFs) zu einer nachhaltigen Hemmung der m*HTT*-Expression in Fibroblasten und Neuronen von HK-Patienten führten, ohne die Expression des normalen *HTT*-Allels oder anderer Gene, die nichtexpandierte CAG-Repeats beinhalten, zu beeinflussen. Zudem konnte ein ZFP mit aktivem Repressorelement nach intrastriataler Injektion in R6/2-Mäusen selektiv die mHTT-Spiegel senken und Verbesserungen im klinischen Phänotyp hervorrufen [87]. Takeda und Sangamo arbeiten gemeinsam an der Entwicklung eines allelselektiven ZFP-TFs zur Transkriptionsmodulation.

#### *HTT*-Inaktivierung mittels CRISPR/Cas9

CRISPR/Cas9 ist eine innovative Methode zur Modifikation des mutierten *HTT*. Zunächst als Abwehrmechanismus gegen Viren beschrieben [36] kann CRISPR/Cas9 auch zur Genomeditierung verwendet werden [14]. Zur Inaktivierung eines Gens oder Editierung spezifischer Gensequenzen werden eine Leit-RNA-Sequenz („single guide RNA“, sgRNA) und das Enzym Cas9 eingesetzt. Die Leit-RNA bindet und rekrutiert das Cas9-Enzym an eine spezifische DNA-Zielsequenz, wo es einen DNA-Doppelstrangbruch (dsDNA) verursacht und die dsDNA-Reparaturmaschinerie aktiviert [14]. Studien mit Fibroblasten von HK-Patienten konnten nachweisen, dass CRISPR/Cas9 das mutierte *HTT*-Allel selektiv und dauerhaft inaktivieren und die mHTT-Produktion verhindern kann [13, 16, 55]. Zudem zeigten Studien eine Verbesserung des Verhaltens und der Neuropathologie transgener Mäusen sowie eine vollständige allelselektive [55] bzw. nichtallelselektive [86] *HTT*-Inaktivierung nach intrakranieller Verabreichung eines CRISPR/Cas9 Systems mittels viraler Vektoren.

Trotz dieser vielversprechenden Ergebnisse sind viele Anwendungsfragen (effektiv erreichtes Verteilungsvolumen im Gehirn nach Gentherapie?) sowie wichtige Sicherheitsfragen (DNA-Schädigungen durch CRISPR/Cas9? Immunvermittelte Reaktionen auf die Präsenz eines bakteriellen Proteins?) noch ungeklärt.

### Modifikation der somatischen Instabilität

**Figure 2:**
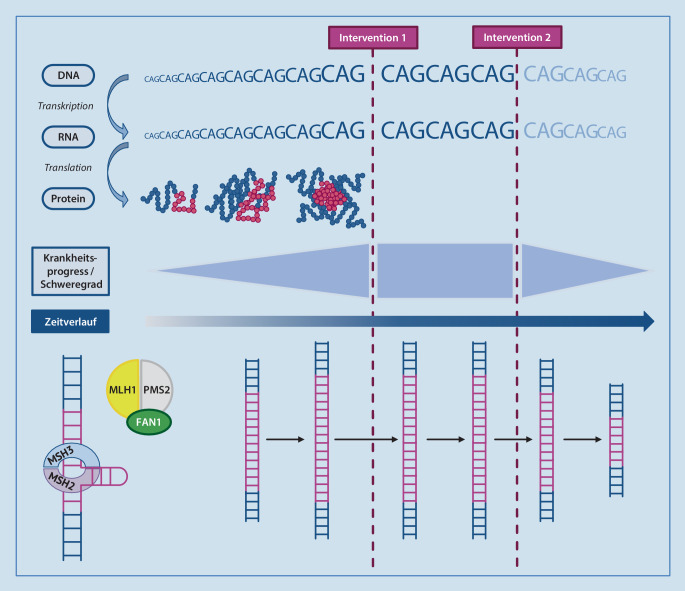

Unter der Annahme, dass die HK-Pathogenese maßgeblich durch die instabile, expandierte CAG-Wiederholung im *HTT* beeinflusst wird, wäre ein innovativer Ansatz zur Krankheitsmodifikation, die CAG-Repeatlänge bzw. deren Zunahme über die Zeit selbst zu manipulieren ([49]; Abb. 2). Man geht davon aus, dass der Krankheitsbeginn zu etwa 60 % durch die Anzahl der CAG-Wiederholungen erklärt werden kann, wobei eine inverse Korrelation zwischen CAG-Repeatlänge und Krankheitsbeginn besteht [4]. Dabei muss zwischen der Instablität auf Keimbahnebene und der somatischen Instabilität der pathologisch verlängerten CAG-Sequenz unterschieden werden. Intergenerationell kann es zu einer Expansion der instabilen CAG-Sequenzwiederholung kommen und damit in der nachfolgenden Generation zu einem früheren Symptombeginn und schnelleren Krankheitsverlauf führen („genetische Antizipation“). Eine genetische Antizipation tritt im Falle der HK bei paternaler Vererbung häufiger und ausgeprägter auf als bei maternaler. Post-mortem-Untersuchungen an humanen und murinen Gehirnproben konnten zudem eine deutliche Zunahme der CAG-Länge im Körpergewebe (v. a. in Nervenzellen) nachweisen („somatische Expansion“), die mit dem Schweregrad der Erkrankung korrelieren [69]. Die instabile CAG-Expansion scheint somit auch den Krankheitsverlauf der Patienten zu modulieren. In genomweiten Assoziationsstudien konnten zudem Gene, die für Elemente der zelleigenen DNA-Reparatur codieren, als genetische Modifikatoren des Krankheitsbeginns und der Krankheitsprogressionsrate identifiziert werden [24, 25].

Eine Hemmung der somatischen Expansion durch Modulation der neuronalen DNA-Reparaturmaschinerie ist somit ein vielversprechender Ansatz, um den Krankheitsverlauf in verschiedenen Stadien zu modulieren. Die Idee hierbei ist, die DNA-Reparaturmaschinerie so zu modifizieren, dass die somatische Expansion reduziert wird [24]. Erste präklinische Studien deuten darauf hin, dass eine Reduktion der *MSH3*-Expression [21] oder eine Steigerung der *FAN1*-Expression [26] die somatische Expansion des m*HTT*-CAG-Repeats hemmen könnte. Da dieser Ansatz auf die Modifikation einer instabilen CAG-Expansion ausgerichtet ist, stellt er nicht nur für die HK, sondern auch für andere Trinukleotidexpansionserkrankungen (z. B. verschiedene spinozerebelläre Ataxien [SCAs]) eine vielversprechende therapeutische Strategie dar. Die Forschung auf diesem Gebiet wird u. a. von Triplet Therapeutics vorangetrieben, die Mitte 2020 eine Natural-History-Studie zur Erhebung von Längsschnittdaten zur somatischen Instabilität bei HK-Genträgern begonnen hat (Shield-HD, NCT04406636).

## Schlussbetrachtung

Entsprechend dem wachsenden Verständnis der Pathogenese der HK wurden zunehmend verfeinerte Therapieansätze für die HK entwickelt. Standen zunächst „generische“ Behandlungen, die an der (hypothetischen) gemeinsamen Endstrecke neurodegenerativer Erkrankungen ansetzten, im Vordergrund, wird zunehmend versucht, Prozesse am Beginn der pathophysiologischen Ereigniskette zu beeinflussen. Im Tierversuch zeigen „Gene-silencing“-Ansätze Wirkungen; bei HK-Kranken zeigen sich Biomarker-Effekte [77]. Noch ist es allerdings eine offene Frage, ob sich die erhoffte Verlangsamung der Krankheitsprogression tatsächlich einstellt und in RCTs nachweisen lässt. Zudem bleibt es eine offene Frage, in welcher Phase der Erkrankung die Behandlung am besten eingesetzt werden sollte, um den natürlichen Krankheitsverlauf substanziell und in klinisch relevanter Weise zu verändern. Es ist ungeklärt, ob und inwieweit die im Krankheitsverlauf bereits entstandenen Schäden repariert werden können, wenn die Körperzellen vom schädigenden Einfluss mutanter Huntingtin-Genprodukte entlastet sind.

## Fazit für die Praxis

- Gegenwärtig ist für keine der hier diskutierten potenziell krankheitsmodifizierenden Therapieansätze ein klinischer Wirksamkeitsnachweis erbracht.
- Trotz Rückschlägen gibt die Vielzahl innovativer, krankheitsspezifischer Therapieansätze Anlass zur Hoffnung, die bislang unaufhaltsame Progression der Huntington-Krankheit (HK) in absehbarer Zeit verlangsamen zu können.
- In den Vordergrund gerückt sind krankheitsspezifische Therapieansätze, die proximal in der Kette der pathophysiologischen Ereignisse oder an der Mutation selbst ansetzen.
- Für Therapien, die eine Modulation der (hypothetischen) gemeinsamen Endstrecke neurodegenerativer Erkrankungen zum Ziel haben, sind bisher keine klinischen Wirksamkeitsnachweise im Sinne einer Verlangsamung der Krankheitsprogression gelungen.
- Noch bleibt die multidisziplinäre, stadiengerechte neuropsychiatrische Versorgung der Huntington-Kranken und ihrer Familien mithilfe zunehmend verfeinerter pharmakologischer und nichtpharmakologischer Strategien zur Symptomkontrolle und Funktionsoptimierung die wichtigste praktische Aufgabe für die Versorger.

## References

[1] Albin RL, Young AB, Penney JB (1995). The functional anatomy of disorders of the basal ganglia. Trends Neurosci.

[2] Alterman JF, Godinho B, Hassler MR (2019). A divalent siRNA chemical scaffold for potent and sustained modulation of gene expression throughout the central nervous system. Nat Biotechnol.

[3] Amaro IA, Henderson LA (2016). An intrabody drug (rAAV6-INT41) reduces the binding of N-terminal Huntingtin fragment(s) to DNA to basal levels in PC12 cells and delays cognitive loss in the R6/2 animal model. J Neurodegener Dis.

[4] Andrew SE, Goldberg YP, Kremer B (1993). The relationship between trinucleotide (CAG) repeat length and clinical features of Huntington’s disease. Nat Genet.

[5] Bañez-Coronel M, Ayhan F, Tarabochia AD (2015). RAN translation in Huntington disease. Neuron.

[6] Beal MF (1994). Neurochemistry and toxin models in Huntington’s disease. Curr Opin Neurol.

[7] Beal MF, Brouillet E, Jenkins BG (1993). Neurochemical and histologic characterization of striatal excitotoxic lesions produced by the mitochondrial toxin 3-nitropropionic acid. J Neurosci.

[8] Beal MF, Kowall NW, Ellison DW (1986). Replication of the neurochemical characteristics of Huntington’s disease by quinolinic acid. Nature.

[9] Behrens PF, Franz P, Woodman B (2002). Impaired glutamate transport and glutamate-glutamine cycling: downstream effects of the Huntington mutation. Brain.

[10] Biogen (2018) Spinraza® (Nusinersen): Berichte über das Auftreten eines kommunizierenden Hydrozephalus, der nicht mit einer Meningitis oder Blutung in Verbindung steht. https://www.bfarm.de/SharedDocs/Risikoinformationen/Pharmakovigilanz/DE/RHB/2018/rhb-spinraza.pdf;jsessionid=E2DE942095F1AEF35D7B5CD2D4601FA8.internet571?__blob=publicationFile. Zugegriffen: 5. Okt. 2021

[11] Cambon K, Zimmer V, Martineau S (2017). Preclinical evaluation of a lentiviral vector for Huntingtin silencing. Mol. Ther. Methods. Clin. Dev..

[12] Cankurtaran ES, Ozalp E, Soygur H (2006). Clinical experience with risperidone and memantine in the treatment of Huntington’s disease. J Natl Med Assoc.

[13] Caron NS, Dorsey ER, Hayden MR (2018). Therapeutic approaches to Huntington disease: from the bench to the clinic. Nat Rev Drug Discov.

[14] Cong L, Ran FA, Cox D (2013). Multiplex genome engineering using CRISPR/Cas systems. Science.

[15] Coyle JT, Schwarcz R (1976). Lesion of striatal neurones with kainic acid provides a model for Huntington’s chorea. Nature.

[16] Dabrowska M, Juzwa W, Krzyzosiak WJ (2018). Precise excision of the CAG tract from the Huntingtin gene by Cas9 nickases. Front Neurosci.

[17] Davies SW, Turmaine M, Cozens BA (1997). Formation of neuronal intranuclear inclusions underlies the neurological dysfunction in mice transgenic for the HD mutation. Cell.

[18] Difiglia M, Sapp E, Chase KO (1997). Aggregation of Huntingtin in neuronal Intranuclear inclusions and dystrophic neurites in brain. Science.

[19] Evers MM, Miniarikova J, Juhas S (2018). AAV5-miHTT gene therapy demonstrates broad distribution and strong human mutant Huntingtin lowering in a Huntington’s disease minipig model. Mol Ther.

[20] Ferreira JJ, Rosser A, Craufurd D (2015). Ethyl-eicosapentaenoic acid treatment in Huntington’s disease: a placebo-controlled clinical trial. Mov Disord.

[21] Flower M, Lomeikaite V, Ciosi M (2019). MSH3 modifies somatic instability and disease severity in Huntington’s and myotonic dystrophy type 1. Brain.

[22] Foroud T, Gray J, Ivashina J (1999). Differences in duration of Huntington’s disease based on age at onset. J Neurol Neurosurg Psychiatry.

[23] Geary RS, Norris D, Yu R (2015). Pharmacokinetics, biodistribution and cell uptake of antisense oligonucleotides. Adv Drug Deliv Rev.

[24] Genetic Modifiers of Huntington’s Disease (Gem-Hd) Consortium (2019). CAG repeat not polyglutamine length determines timing of Huntington’s disease onset. Cell.

[25] Genetic Modifiers of Huntington’s Disease (Gem-Hd) Consortium (2015). Identification of genetic factors that modify clinical onset of Huntington’s disease. Cell.

[26] Goold R, Flower M, Moss DH (2019). FAN1 modifies Huntington’s disease progression by stabilizing the expanded HTT CAG repeat. Hum Mol Genet.

[27] Guo Q, Bin H, Cheng J (2018). The cryo-electron microscopy structure of huntingtin. Nature.

[28] Harding RJ, Tong YF (2018). Proteostasis in Huntington’s disease: disease mechanisms and therapeutic opportunities. Acta Pharmacol Sin.

[29] Hersch SM, Gevorkian S, Marder K (2006). Creatine in Huntington disease is safe, tolerable, bioavailable in brain and reduces serum 8OH2′dG. Neurology.

[30] Hersch SM, Schifitto G, Oakes D (2017). The CREST-E study of creatine for Huntington disease: a randomized controlled trial. Neurology.

[31] Huntington Study Group (2003). Dosage effects of riluzole in Huntington’s disease: a multicenter placebo-controlled study. Neurology.

[32] Huntington Study Group (2004). Minocycline safety and tolerability in Huntington disease. Neurology.

[33] Huntington Study Group (2001). A randomized, placebo-controlled trial of coenzyme Q10 and remacemide in Huntington’s disease. Neurology.

[34] Huntington Study Group TREND-HD Investigators (2008). Randomized controlled trial of ethyl-eicosapentaenoic acid in Huntington disease: the TREND-HD study. Arch Neurol.

[35] Huntington’s Disease Collaborative Research Group (1993). A novel gene containing a trinucleotide repeat that is expanded and unstable on Huntington’s disease chromosomes. Cell.

[36] Jinek M, Chylinski K, Fonfara I (2012). A programmable dual-RNA-guided DNA endonuclease in adaptive bacterial immunity. Science.

[37] Jones L, Houlden H, Tabrizi SJ (2017). DNA repair in the trinucleotide repeat disorders. Lancet Neurol.

[38] Keskin S, Brouwers CC, Sogorb-Gonzalez M (2019). AAV5-miHTT lowers Huntingtin mRNA and protein without off-target effects in patient-derived neuronal cultures and astrocytes. Mol Ther Methods Clin Dev.

[39] Keum JW, Shin A, Gillis T (2016). The HTT CAG-expansion mutation determines age at death but not disease duration in Huntington disease. Am J Hum Genet.

[40] Khan E, Mishra SK, Mishra R (2019). Discovery of a potent small molecule inhibiting Huntington’s disease (HD) pathogenesis via targeting CAG repeats RNA and poly Q protein. Sci Rep.

[41] Kingwell K (2021). Double setback for ASO trials in Huntington disease. Nat Rev Drug Discov.

[42] Kordasiewicz HB, Stanek LM, Wancewicz EV (2012). Sustained therapeutic reversal of Huntington’s disease by transient repression of huntingtin synthesis. Neuron.

[43] Kremer B, Clark CM, Almqvist EW (1999). Influence of lamotrigine on progression of early Huntington disease: a randomized clinical trial. Neurology.

[44] Küppenbender KD, Standaert DG, Feuerstein TJ (2000). Expression of NMDA receptor subunit mRNAs in neurochemically identified projection and interneurons in the human striatum. J Comp Neurol.

[45] Kurosaki T, Popp MW, Maquat LE (2019). Quality and quantity control of gene expression by nonsense-mediated mRNA decay. Nat Rev Mol Cell Biol.

[46] Landwehrmeyer GB, Dubois B, de Yébenes JG (2007). Riluzole in Huntington’s disease: a 3-year, randomized controlled study. Ann Neurol.

[47] Landwehrmeyer GB, Standaert DG, Testa CM (1995). NMDA receptor subunit mRNA expression by projection neurons and interneurons in rat striatum. J Neurosci.

[48] Leavitt BR, Tabrizi SJ (2020). Antisense oligonucleotides for neurodegeneration. Science.

[49] López Castel A, Cleary JD, Pearson CE (2010). Repeat instability as the basis for human diseases and as a potential target for therapy. Nat Rev Mol Cell Biol.

[50] Lucetti C, Del Dotto P, Gambaccini G (2003). IV amantadine improves chorea in Huntington’s disease: an acute randomized, controlled study. Neurology.

[51] Ludolph AC, He F, Spencer PS (1991). 3-Nitropropionic acid-exogenous animal neurotoxin and possible human striatal toxin. Can J Neurol Sci.

[52] Mcgarry A, Leinonen M, Kieburtz K (2020). Effects of pridopidine on functional capacity in early-stage participants from the PRIDE-HD study. J Huntingtons Dis.

[53] Mcgarry A, Mcdermott M, Kieburtz K (2017). A randomized, double-blind, placebo-controlled trial of coenzyme Q10 in Huntington disease. Neurology.

[54] Miniarikova J, Zanella I, Huseinovic A (2016). Design, characterization, and lead selection of therapeutic miRNas targeting Huntingtin for development of gene therapy for Huntington’s disease. Mol Ther Nucleic Acids.

[55] Monteys AM, Ebanks SA, Keiser MS (2017). CRISPR/Cas9 editing of the mutant Huntingtin allele in vitro and in vivo. Mol Ther.

[56] Novartis (2020) Novartis receives US food and drug administration (FDA) orphan drug designation for branaplam (LMI070) in Huntington’s disease (HD). https://www.novartis.com/news/media-releases/novartis-receives-us-food-and-drug-administration-fda-orphan-drug-designation-branaplam-lmi070-huntington%27s-disease-hd. Zugegriffen: 31. Juli 2021

[57] Okamoto S, Pouladi MA, Talantova M (2009). Balance between synaptic versus extrasynaptic NMDA receptor activity influences inclusions and neurotoxicity of mutant huntingtin. Nat Med.

[58] Pearce MMP, Spartz EJ, Hong W (2015). Prion-like transmission of neuronal huntingtin aggregates to phagocytic glia in the drosophila brain. Nat Commun.

[59] Perry TL, Hansen S (1990). What excitotoxin kills striatal neurons in Huntington’s disease? Clues from neurochemical studies. Neurology.

[60] Perry TL, Hansen S, Kloster M (1973). Huntington’s chorea. Deficiency of gamma-aminobutyric acid in brain. N Engl J Med.

[61] Pfister EL, Kennington L, Straubhaar J (2009). Five siRNAs targeting three SNPs may provide therapy for three-quarters of Huntington’s disease patients. Curr Biol.

[62] Puri BK, Leavitt BR, Hayden MR (2005). Ethyl-EPA in Huntington disease: a double-blind, randomized, placebo-controlled trial. Neurology.

[63] Rawlins MD, Wexler NS, Wexler AR (2016). The prevalence of Huntington’s disease. Neuroepidemiology.

[64] Reilmann R, Gordon MF, Anderson KE (2019). The efficacy and safety results of laquinimod as a treatment for Huntington disease (LEGATO-HD). Neurology.

[65] Reilmann R, Mcgarry A, Grachev ID (2019). Safety and efficacy of pridopidine in patients with Huntington’s disease (PRIDE-HD): a phase 2, randomised, placebo-controlled, multicentre, dose-ranging study. Lancet Neurol.

[66] Roche (2021) Roche provides update on tominersen programme in manifest Huntington’s disease. https://www.roche.com/dam/jcr:e077be26-41a0-4431-ae19-8f8dc846179a/en/22032021-mr-update-on-tominersen-programme-en.pdf. Zugegriffen: 31. Juli 2021

[67] Sanberg PR, Coyle JT (1984). Scientific approaches to Huntington’s disease. CRC Crit Rev Clin Neurobiol.

[68] Sapp E, Kegel KB, Aronin N (2001). Early and progressive accumulation of reactive microglia in the Huntington disease brain. J Neuropathol Exp Neurol.

[69] Shelbourne PF, Keller-Mcgandy C, Bi WL (2007). Triplet repeat mutation length gains correlate with cell-type specific vulnerability in Huntington disease brain. Hum Mol Genet.

[70] Shenkman M, Geva M, Gershoni-Emek N (2021). Pridopidine reduces mutant huntingtin-induced endoplasmic reticulum stress by modulation of the sigma-1 receptor. J Neurochem.

[71] Shoulson I, Odoroff C, Oakes D (1989). A controlled clinical trial of baclofen as protective therapy in early Huntington’s disease. Ann Neurol.

[72] Silva AC, Lobo DD, Martins IM (2019). Antisense oligonucleotide therapeutics in neurodegenerative diseases: the case of polyglutamine disorders. Brain.

[73] Spronck EA, Vallès A, Lampen MH (2021). Intrastriatal administration of AAV5-miHTT in non-human primates and rats is well tolerated and results in miHTT transgene expression in key areas of Huntington disease pathology. Brain Sci.

[74] Stanek LM, Sardi SP, Mastis B (2014). Silencing mutant Huntingtin by adeno-associated virus-mediated RNA interference ameliorates disease manifestations in the YAC128 mouse model of Huntington’s disease. Hum Gene Ther.

[75] Stoker TB, Andresen KER, Barker RA (2021). Hydrocephalus complicating intrathecal antisense oligonucleotide therapy for Huntington’s disease. Mov Disord.

[76] Tabrizi SJ, Ghosh R, Leavitt BR (2019). Huntingtin lowering strategies for disease modification in Huntington’s disease. Neuron.

[77] Tabrizi SJ, Leavitt BR, Landwehrmeyer GB (2019). Targeting Huntingtin expression in patients with Huntington’s disease. N Engl J Med.

[78] Taylor-Robinson SD, Weeks RA, Bryant DJ (1996). Proton magnetic resonance spectroscopy in Huntington’s disease: evidence in favour of the glutamate excitotoxic theory. Mov Disord.

[79] Tomoshige S, Nomura S, Ohgane K (2017). Discovery of small molecules that induce the degradation of Huntingtin. Angew. Chem. Int. Ed. Engl..

[80] Túnez I, Tasset I, Pérez-De La Cruz V (2010). 3-Nitropropionic acid as a tool to study the mechanisms involved in Huntington’s disease: past, present and future. Molecules.

[81] Vaccinex Inc. (2020) Top-line results of phase 2 SIGNAL study in Huntington’s disease support potential for cognitive benefit of pepinemab. https://ir.vaccinex.com/news-releases/news-release-details/top-line-results-phase-2-signal-study-huntingtons-disease/. Zugegriffen: 31. Juli 2021

[82] Verny C, Bachoud-Lévi AC, Durr A (2017). A randomized, double-blind, placebo-controlled trial evaluating cysteamine in Huntington’s disease. Mov Disord.

[83] Vonsattel JP, Keller C, Cortes Ramirez EP (2011). Huntington’s disease—neuropathology. Handb Clin Neurol.

[84] Wave Life Sciences Ltd. (2021) Wave life sciences provides update on phase 1b/2a PRECISION-HD trials. https://ir.wavelifesciences.com/news-releases/news-release-details/wave-life-sciences-provides-update-phase-1b2a-precision-hd. Zugegriffen: 31. Juli 2021

[85] Wild EJ, Tabrizi SJ (2017). Therapies targeting DNA and RNA in Huntington’s disease. Lancet Neurol.

[86] Yang S, Chang R, Yang H (2017). CRISPR/Cas9-mediated gene editing ameliorates neurotoxicity in mouse model of Huntington’s disease. J Clin Invest.

[87] Zeitler B, Froelich S, Marlen K (2019). Allele-selective transcriptional repression of mutant HTT for the treatment of Huntington’s disease. Nat Med.

[88] Zhao T, Hong Y, Li XJ (2016). Subcellular clearance and accumulation of Huntington disease protein: a mini-review. Front Mol Neurosci.

